# Trends in Breast Cancer Screening in a Safety-Net Hospital During the COVID-19 Pandemic

**DOI:** 10.1001/jamanetworkopen.2021.19929

**Published:** 2021-08-06

**Authors:** Ana I. Velazquez, Jessica H. Hayward, Blake Gregory, Niharika Dixit

**Affiliations:** 1 Division of Hematology/Oncology, Department of Medicine, University of California, San Francisco; 2 National Clinician Scholars Program, Philip R. Lee Institute for Health Policy Studies, University of California, San Francisco; 3 Department of Radiology and Biomedical Imaging, University of California, San Francisco; 4 San Francisco Health Network, San Francisco, California; 5 Division of Hematology/Oncology, Department of Medicine, Zuckerberg San Francisco General Hospital, San Francisco, California

## Abstract

This cross-sectional study uses electronic health record data to evaluate the association between COVID-19 and breast cancer screening at an urban integrated health system’s safety-net hospital between September 2019 and January 2021.

## Introduction

National estimates project COVID-19 negatively influenced cancer screening, leading to an estimated deficit of 3.9 million breast cancer (BC) screenings among US adults.^1,2^ In San Francisco, California, low-income neighborhoods disproportionately affected by COVID-19 bear the burden of higher BC stage at diagnosis.^3,4^ We sought to evaluate the association of COVID-19 and BC screening in a safety-net hospital in San Francisco.

## Methods

This cross-sectional study evaluated trends in BC screening at an urban integrated health system’s safety-net hospital. We obtained the number of screening mammograms per month during 2019 from electronic health record (EHR) data, and aggregate numbers between September 1, 2019, and January 31, 2021, after the implementation of a new EHR. The number of screening mammograms per month was plotted against the 2019 baseline. Proportions of completed tests by phase of the pandemic (pre–COVID-19, first stay-at-home order, reopening, and second stay-at-home order) were compared by race/ethnicity and age with 2-sided, 2-sample proportion tests. Race/ethnicity was used as a proxy for the disproportionate burden of COVID-19 and experiences of individual and systemic racism experienced by minority communities. Analyses were conducted with Stata, version 16 (StataCorp LLC). *P* < .05 was used to determine significance. We followed the Strengthening the Reporting of Observational Studies in Epidemiology (STROBE) reporting guideline for cross-sectional studies.^5^ Deidentified data collected for quality improvement activities does not require approval from The University of California, San Francisco institutional review board; this study was therefore exempted from review.

## Results

**Figure 1.**
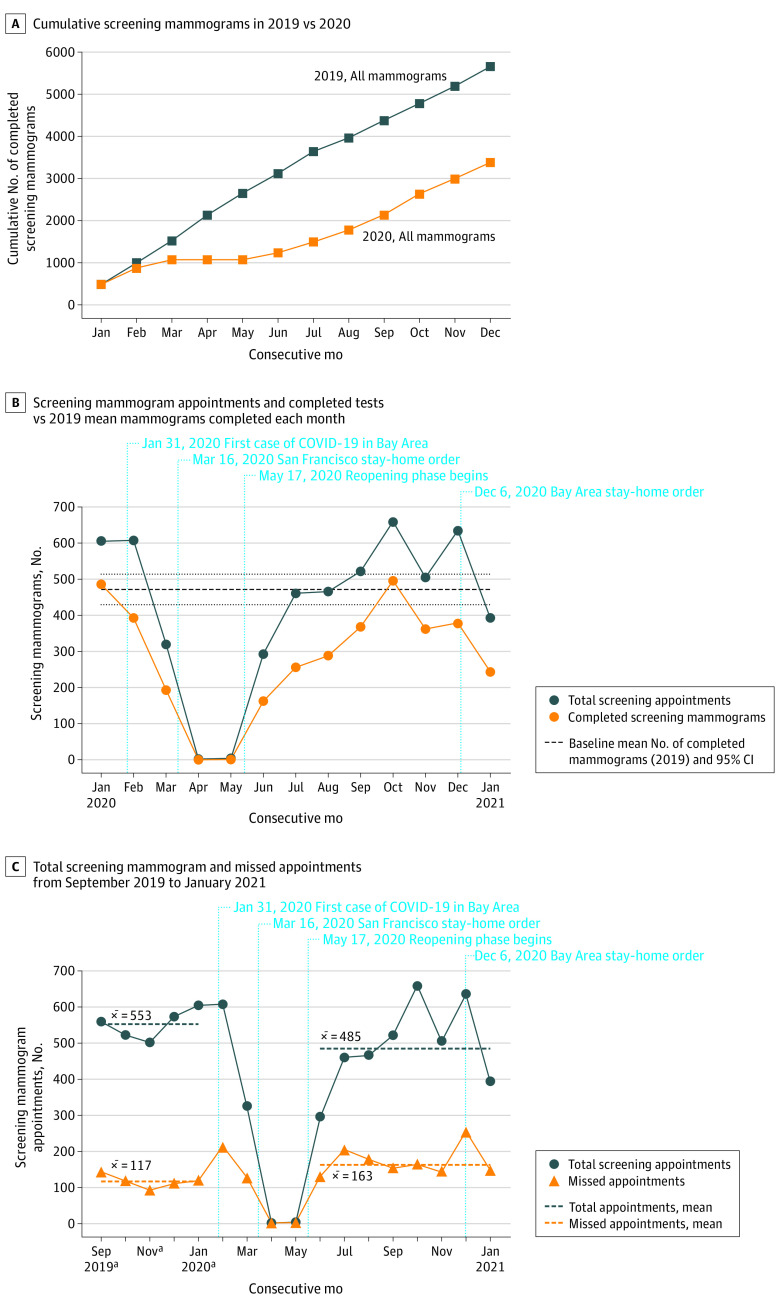
Trends in Number of Screening Mammograms Completed During the COVID-19 Pandemic at an Inner-City Safety-Net Hospital The term *x̅* represents the mean number of total appointments or missed appointments during pre–COVID-19 (September 2019 to January 2020) and the reopening phase (June to November 2020). ^a^The pre–COVID-19 phase was defined as September 2019 to January 2020.

A total of 9291 screening mammograms were performed from January 1, 2019, to January 31, 2021: 5662 during 2019, with a mean of 472 mammograms per month (95% CI, 430-514 mammograms), and 3385 in 2020 (60% of the 2019 volume) (Figure 1A). During the first stay-at-home order (February 1, 2020, to May 31, 2020), the number of screening mammograms decreased to 194 in March (41% of baseline [mean of 472 mammograms per month during 2019]) and to 0 in April (Figure 1B). The number of missed appointments increased during 2020, with 127 of 321 (40%) missed in March, compared with 585 of 2764 (21%) from September 2019 to January 2020 (pre–COVID-19) (Figure 1C). During the reopening phase (June 1, 2020, to November 30, 2020), the number of screening mammograms increased but remained below baseline, except in October, when 496 mammograms were completed. The number of screening mammograms declined during the second stay-at-home order. The mobile mammography unit volume decreased from 831 during 2019 to 248 in 2020, with 0 mammograms between April and June 2020.

**Figure 2.**
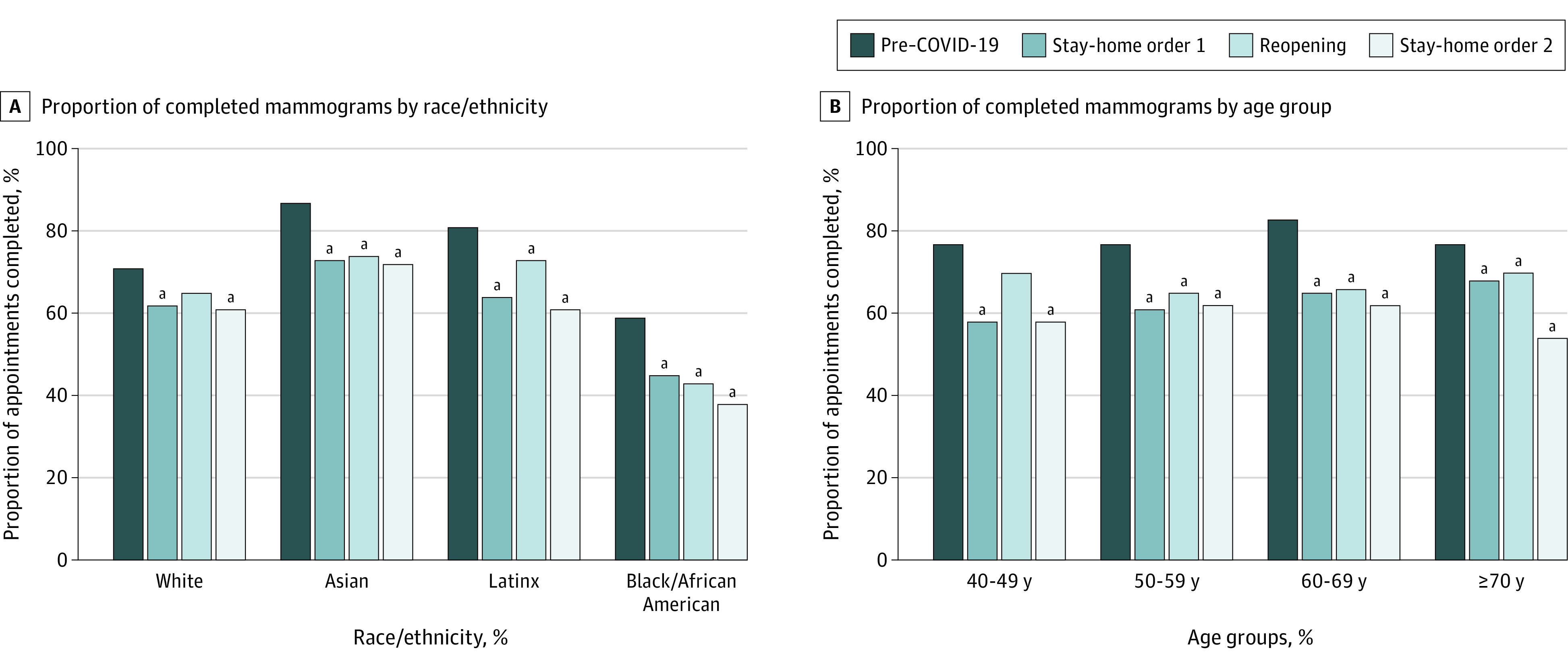
Differences in Proportions of Completed Screening Mammograms During the Different Phases of the COVID-19 Pandemic by Race/Ethnicity and Age Groups Phases of the COVID-19 pandemic were defined to concur with the implementation of local regulations: pre–COVID-19 from September 2019 to January 2020, stay-at-home order 1 from February to May 2020, reopening from June to November 2020, and stay-at-home order 2 from December 2020 to January 2021. Proportions were calculated with the number of completed tests as numerator over the sum of the number of missed appointments and completed tests as denominator. ^a^Statistically significant difference in proportions when compared with pre–COVID-19 for the same racial/ethnic or age group (*P* < .05 for the test of proportions).

The proportion of monthly screening mammograms completed was 244 of 392 scheduled (62%) in January 2021 compared with a mean (SD) of 436 (36) of 553 (41) (79%) from September 2019 to January 2020 (pre–COVID-19; test of proportions *P* < .001). Compared with pre–COVID-19, the volume and proportion of mammograms completed decreased across racial/ethnic groups (from 311 of 441 [71%] pre–COVID-19 to 96 of 157 [61%] during the second stay-at-home order among White women; from 941 of 1081 [87%] to 220 of 307 [72%] among Asian women; from 716 of 881 [81%] to 230 of 378 [61%] among Latinx women; and from 203 of 344 [59%] to 70 of 184 [38%] among Black/African American women) and age groups (from 241 of 312 [77%] to 63 of 108 [58%] among women 40-49 years; from 774 of 1011 [77%] to 266 of 427 [62%] among women 50-59 years; from 861 of 1043 [83%] to 230 of 371 [62%] among women 60-69 years; and from 290 of 376 [77%] to 64 of 119 [54%] among women ≥70 years) (Figure 2). The proportion of completed mammograms was lowest among Black women at all time points, younger women during the first stay-at-home order, and women aged 70 years or older during the second stay-at-home order.

## Discussion

The reduction in the cumulative number of mammograms suggests a substantial deficit of missed BC screening, which may worsen preexisting disparities. Our results are consistent with those of reports that found discontinuation of BC screening in April 2020.^2,6^ In contrast to reports showing recovery of screening volumes,^2,6^ our data highlight persistent low BC screening volumes and an absolute decrease in the proportion of completed mammograms among Latinx and Black women. We hypothesize that these differences by race/ethnicity are multilevel and reflect the effect of worry, competing priorities, limited access, and disproportionate burden and socioeconomic impact of COVID-19 in Latinx and Black communities.^4^ Resource and staff reallocation from preventive health community-based efforts likely contributes to these disparities, as suggested by the lower patient volumes of our mobile unit.

Limitations of this study include the use of aggregate data from a single institution, use of race/ethnicity as recorded in the EHR, and lack of baseline characteristics before August 2019. Although vaccination efforts are a top priority, health care systems should leverage COVID-19–related community outreach and engagement to develop concerted efforts that promote preventive care and ensure preexisting disparities do not worsen among communities with higher risk.
